# Linker Design Impacts Antibody-Drug Conjugate Pharmacokinetics and Efficacy *via* Modulating the Stability and Payload Release Efficiency

**DOI:** 10.3389/fphar.2021.687926

**Published:** 2021-06-23

**Authors:** Dian Su, Donglu Zhang

**Affiliations:** Drug Metabolism and Pharmacokinetics, Genentech, Inc., South San Francisco, CA, United States

**Keywords:** linker design, stability, payload release, immolation, threshold dose, dose regimen, antibody-drug conjugate

## Abstract

The development of antibody-drug conjugates (ADCs) has significantly been advanced in the past decade given the improvement of payloads, linkers and conjugation methods. In particular, linker design plays a critical role in modulating ADC stability in the systemic circulation and payload release efficiency in the tumors, which thus affects ADC pharmacokinetic (PK), efficacy and toxicity profiles. Previously, we have investigated key linker parameters such as conjugation chemistry (e.g., maleimide vs. disulfide), linker length and linker steric hindrance and their impacts on PK and efficacy profiles. Herein, we discuss our perspectives on development of integrated strategies for linker design to achieve a balance between ADC stability and payload release efficiency for desired efficacy in antigen-expressing xenograft models. The strategies have been successfully applied to the design of site-specific THIOMAB^TM^ antibody-drug conjugates (TDCs) with different payloads. We also propose to conduct dose fractionation studies to gain guidance for optimal dosing regimens of ADCs in pre-clinical models.

## Introduction

To date, ten antibody-drug conjugates (ADCs) have been approved by the Food and Drug Administration (FDA) with four also approved by the European Medicines Agency (EMA) (Hafeez et al., 2020; Joubert et al., 2020). Recent approvals of Zynlonta™ (loncastuximab tesirine-lpyl), Trodelvy^®^ (sacituzumab govitecan-hziy), Polivy^®^ (polatuximab vendotin) Enhertu^®^ (trastuzumab deruxtecan), Adcetris^®^ (brentuximab vendotin), Kadcyla^®^ (ado-trastuzumab emtansine), and Besponsa^®^ (inotuzumab ozogamicin) have increased interest in expanding the applications of ADCs. ADCs consist of an antibody that targets a disease-associated antigen(s) and a payload drug that is connected to the antibody via a chemical linker. ADC payloads are often potent antimitotic cytotoxins and DNA alkylators as well as agents with other cell-killing mechanisms. The approved ADC drugs utilize both cleavable and non-cleavable linkers that are connected to lysines, inter-chain cysteines, or site-specific cysteines (Joubert et al., 2020; Zhao et al., 2020). Linkers are made of protease-sensitive peptides (e.g., Enhertu^®^ (Ogitani et al., 2016), Polivy^®^ (Deeks, 2019), Adcetris^®^ (Doronina et al., 2003), Zynlonta^TM^ (Zammarchi et al., 2018) or acid-labile components (e.g., Besponsa^®^ (Shor et al., 2015). Recently, stable disulfide-based ADC linkers have been disclosed that decouple plasma stability from payload release in tumors (Chari et al., 1995; Erickson et al., 2006; Pillow et al., 2017). Herein, we focus on discussing influence of linker design on delivery of payloads to the site of action and associated efficacy profiles *via* a balance between ADC systemic stability and intratumoral payload release. The provided perspectives are built upon the overview of our recent publications and the retrospective summary of our key findings.

## Modulating Antibody-Drug Conjugate Stability *via* Conjugation Site, Linker Length, and Linker Steric Hindrance

**FIGURE 1:**
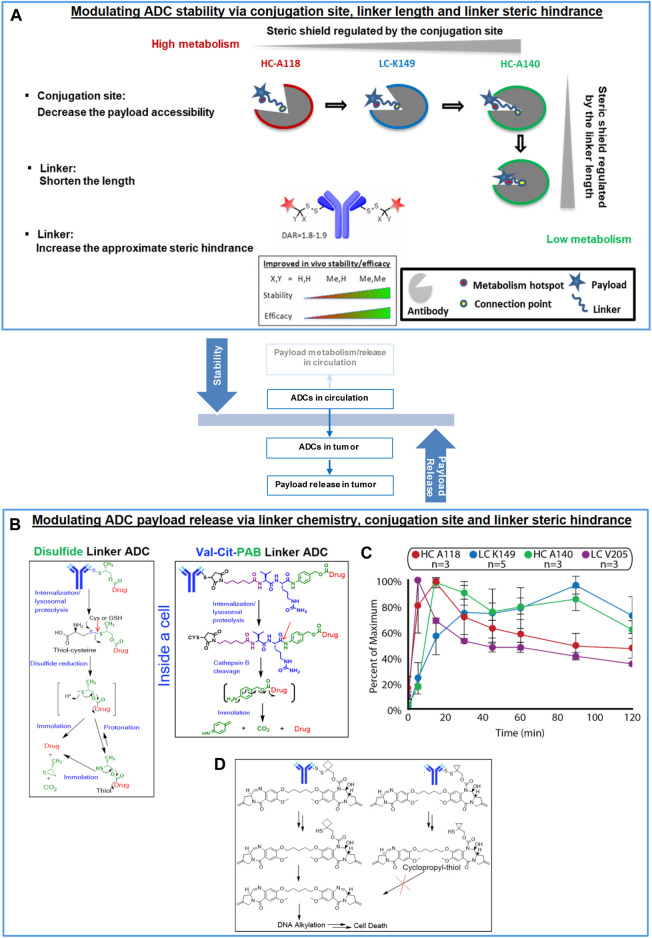
Balancing ADC stability and payload release *via* linker design and conjugation site. **(A)** Modulating ADC stability via conjugation site, linker length and proximal steric hindrance (increased ADC stability by decreasing the payload accessibility, shortening the linker length and increasing the proximal steric hindrance of the linker), **(B)** Mechanisms of linker cleavage and immolation, **(C)** Payload release kinetics in the antibody-conjugated drug assays influenced by conjugation sites (*n* = 3–5 antibodies per conjugation site). Reprinted with permission from (Lee et al., 2020). Copyright (2020) American Chemical Society. **(D)** Comparison of immolation pathways of aCD22-PBD TDCs with cyclobutyl- and cyclopropyl-disulfide linker.

ADCs undergo biotransformation in the systemic circulation and in tissue cells. Linker deconjugation/cleavage, linker immolation and payload metabolism are considered typical major biotransformation pathways (Supplementary Figure S1) besides antibody metabolism and catabolism. From the administration of ADCs into the circulation to the action of released payloads in target cells, diverse catabolites can be generated through biotransformation, ranging from large molecules to small molecules, that are either pharmacologically active or inactive. Ideally, ADCs are desired to stay stable or intact in the circulation prior to entering target cells whereas there are situations where ADC catabolites are still biologically active (Su et al., 2018; Poreba, 2020). In most cases, the metabolic stability reflects the intact ADC level given the similar antibody PK parameters such as clearance, distribution volume and half-life mostly across different linker-drug variants. Therefore, ADC stability is herein primarily refers to the metabolic stability or integrity. A number of approaches involving conjugation site selection and linker modification have been developed to enhance ADC stability (Pillow et al., 2017; Sadowsky et al., 2017; Anami et al., 2018; Su et al., 2018). Generally, modifications can be performed on each component (e.g., antibody, linker, and payload) for such purpose. Our investigation has revealed that conjugation site, linker length, and linker steric hindrance are effective general approaches for site-specific THIOMAB^TM^ ADCs (TDCs) (Figure 1A) and should be more broadly applicable to a variety of ADC platforms (Su et al., 2018). The desired steric shield provided by the antibody can be achieved by selecting a more sterically hindered conjugation or attachment site. On the other hand, an alternative chemical modification to introduce proximal steric hindrance around the cleavable or labile site of the linker has been demonstrated to be an effective method of improving stability (Erickson et al., 2006; Pillow et al., 2017; Sadowsky et al., 2017; Anami et al., 2018) and references therein. ADC biotransformation and drug-antibody-ratio (DAR) profiling have become the essential integrated information for assessing and understanding ADC stability (Xu et al., 2013; Su et al., 2018).

Our previous research suggested that aHER2-MMAE TDC efficacy correlated with systemic stability (Shen et al., 2012). The site choice of conjugation is a primary parameter by which ADC stability and, thus, efficacy can be modulated. Our most recent investigation further elaborated the underlying reason for such relationships and confirmed that steric shield resulting from different conjugation sites was a primary factor for their modulation of ADC stability (Su et al., 2018). The steric shield provided by the antibody can help to reduce linker deconjugation/cleavage as well as payload metabolism. For example, sites HC-A118, LC-K149 and HC-A140 (according to EU numbering), (Ohri et al., 2018), provided enhanced ADC stability with decreased fractional solvent accessibility (FSA) corresponding to increased steric shield around the conjugation site. Based on this, another primary factor, linker length, was then varied to modulate ADC stability through the influence on the steric shield from the distance between antibody and the payload/drug (Su et al., 2018; Su et al., 2019). We discovered that a shorter linker typically results in better ADC stability by tethering the payload further inside the steric shield of the antibody relative to a longer linker. Ideally, the linker that connects the antibody to the drug should be stable in circulation and cleaved enzymatically (by a protease such as cathepsin B) or chemically (by reducing agents such as cysteine and GSH) inside the cell to release the attached payload to act on the intended target (Zhang et al., 2016a; Zhang et al., 2016b; Ponziani et al., 2020; Poreba, 2020) In the absence of linker stability in serum, premature release of the payload can result in systemic toxicity. Whereas the low stability of Trodelvy^®^ bearing an unstable linker seems to avoid this effect with manageable safety likely due to the selected toxicity profile of its topisomerase I inhibitor payload (Bardia et al., 2017; Sahota and Vahdat, 2017; Bardia et al., 2021). On the other hand, inefficient cleavage of the linker inside the cell may not produce the intended antitumor activity (Zhang et al., 2016a; Zhang et al., 2016b; Ma et al., 2016; Poreba, 2020). Different linker chemistry and linker modifications have been explored. Maleimide and disulfide linkages represent two major types of linker chemistry. Maleimide has been widely utilized as an antibody connection modality and has been employed with both cleavable or non-cleavable linkers. Accordingly, the pharmacologically efficacious component could be the payload itself or that with partial or full-length linkers depending on the mechanism of action of the payload (Su et al., 2019; Poreba, 2020). Either for cleavable or non-cleavable linkers, the efficient release of the active component would be the desired result, which can be achieved through the optimal linker design. Disulfide-based antibody connections have recently been developed as a new strategy for the generation of cleavable linkers (Pillow et al., 2017; Sadowsky et al., 2017). For both maleimide and disulfide cleavable linkers, the steric hindrance created by the proximity of the cleavage site is critical and can be introduced by chemical modifications to modulate stability of the conjugate.

Together, conjugation site, linker length, linkage/conjugation chemistry, cleavable/non-cleavable linkage and steric hindrance are the key parameters for consideration to optimize ADC stability. Undesired payload metabolism/release in circulation could lead to reduced efficacy and/or increased toxicity.

## Modulating Antibody-Drug Conjugate Payload Release *via* Linker Chemistry, Conjugation Site, and Linker Steric Hindrance

Payload release is likely the rate-limiting step after internalization of the conjugate into the cell although lysosomal escape can be the next rate-limiting step for some ADCs. For non-cleavable linkers, this mainly happens in the lysosome where the antibody is catabolized or hydrolyzed by proteases into peptides/amino acids (AAs). In these cases, a biologically active catabolite is produced in which the attached payload retains some type of peptide or AA in its chemical structure and a transporter may be needed for payload lysosomal escape (Kinneer et al., 2018). In contrast, cleavable linkers are designed to release the free payload in an unmodified form. These ADCs often employ chemical modalities which undergo self-immolation following cleavage of the linker group in order to release the attached payload. The rate of payload release in these cases therefore depends on both the linker cleavage and the subsequent immolation steps (Figure 1B).

Despite the fact that the apparent stability in circulation can be similar for ADCs with disulfide vs. maleimide peptide linkers, disulfide cleavage and immolation may be slower than that of maleimide peptide linker following disulfide cleavage and enzymatic cleavage, respectively. Given the same linker chemistry, conjugation site impacts the kinetics of payload release as demonstrated by the TDCs with self-immolating disulfide linkers in the bioanalytical assays measuring antibody-conjugated payloads (Figure 1C). For example, a range of payload release kinetics were observed, from faster to slower, with for linkers attached to the LC-V205, HC-A118, HC-A140, and LC-K149 sites. In particular, the site HC-A118C reached maximal pyrrolo[2,1-c][1,4]benzodiazepine-dimer (PBD) payload release (15 min) faster than site LC-K149C (60 min). The conjugation site directly influenced release kinetics, independent of the monoclonal antibody, as observed with ADC stability. Chemical modifications that introduced steric hindrance at the cleavage sites could help enhance ADC stability. In contrast, steric hindrance could also result in slower or ineffective release of payload. A great example is the comparison between the two closely related PBD TDCs with cyclobutyl vs cyclopropyl hindered disulfide linkers (Figure 1D) (Zhang et al., 2016b). The TDC stability is optimal and similar in circulation in mice. However, the cyclobutyl analog was efficacious in mice while the cyclopropyl analog was not. In-depth investigation suggested that effective intratumoral payload concentration was observed with the cyclobutyl but not the cyclopropyl analog. Further *in vitro* mechanistic investigation demonstrated that effective release of active PBD dimer was achieved by the cyclobutyl-containing molecule. However, no self-immolation following disulfide linker cleavage was observed with the cyclopropyl analog and, consequently the active payload alone was not released for biological activity.

In brief, modulating payload release inside the cell is another critical aspect to consider in addition to ADC stability in circulation. Integrated linker design utilizing alternative chemistries, conjugation site, and proximal linker steric hindrance serves as an effective approach to achieve desired payload release efficiency.

## Balancing Antibody-Drug Conjugate Stability and Payload Release *via* Integrated Linker Design for Optimal Intratumoral Payload PK Profiles and Efficacy

Collectively, an integrated linker design approach is required for the generation of optimal ADCs. Such an approach should incorporate multiple parameters including conjugation site, linker length, linker chemistry, cleavable/non-cleavable linkage, and proximal linker steric hindrance, as discussed previously. We have successfully employed this integrated linker design approach and compared TDCs with different payloads (PBD, MMAE, DMx) to find the decision-making endpoints (Zhang et al., 2019).

**FIGURE 2:**
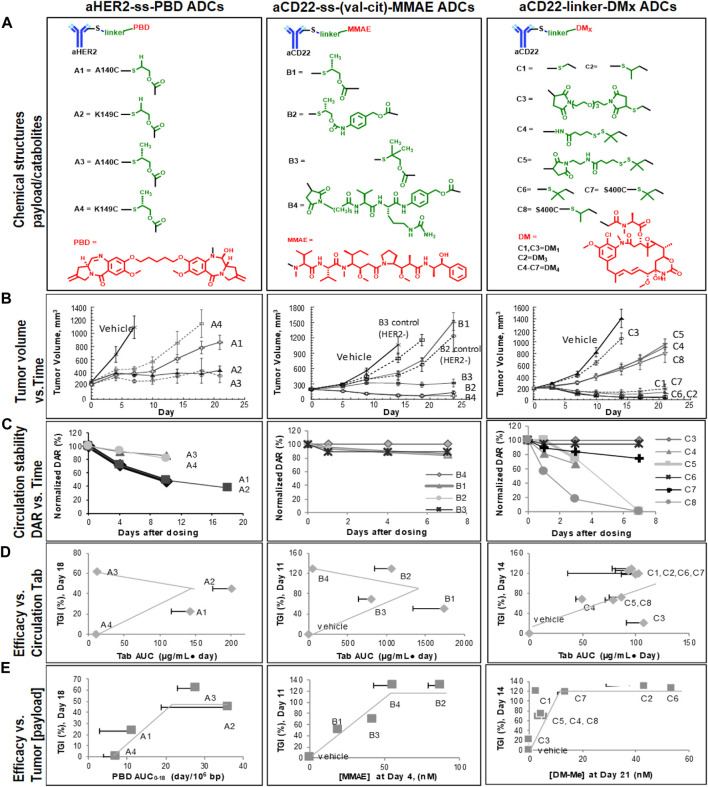
Case studies of PBD-, MMAE-, and DMx-ADCs in mice bearing human HER2-expressing Fo5 or human CD22-expressing BJAB. Luc xenografts (*n* = 8, IV, A1/A2: 4 mg/kg; A3/A4: 0.4 mg/kg; B1-B3: 20 mg/kg; B4: 1 mg/kg; C1-C8: 50 μg/m^2^ or 1–1.6 mg/kg). **(A)** Chemical structures of TDCs and payloads or catabolites, **(B)** Efficacy profiles, **(C)** Normalized DAR and time profiles in plasma, **(D)** Correlation X-Y plots of plasma total antibody AUC exposures (of PBD, MMAE, or DMx conjugates) with TGIrel, and **(E)** Correlation X-Y plots of tumor payload/catabolite exposures (PBD, MMAE, or DMx) with TGIrel. Note: the TGIrel for PBD ADCs were calculated against the least active ADC A4 as apposite to the vehicle group for MMAE and DMx ADCs due to rapid tumor growth and early termination on day 7. Adapted with permission from (Zhang et al., 2019). Copyright 2019 American Society for Pharmacology and Experimental Therapeutics.

As shown in Figure 2A, the relative stability ranking was observed as A1 ∼ A2 < A3 ∼ A4 the aHER2-ss-PBD TDCs. In particular, ADC stability was improved by the enhanced steric hindrance with CH_3_ compared to H flanking the S-S disulfide bond: A1 < A3 (LC-K149C), A2<A4 (HC-A140C), respectively. A4, the most stable PBD TDC based on the DAR profiles, however, turned out to be the least active in the mouse xenograft model *in vivo*. The overall efficacy ranking order was A4 < A1 <**A3/A2** with A2/A3 (in bold) with A2/A3 (in bold) affording meaningful efficacy for tumor stasis. On the other hand, the ranking of the released PBD dimer concentration in tumor A4 < A1 <**A3** <**A2** was roughly in alignment with the observed efficacy trend. In Figure 2B, the DAR profile suggested that aCD22-monomethyl auristatin E (MMAE) TDC stability was similar across different linkers (S-S for B1-B3 vs. maleimide linkage for B4 and different immolation chemistry) at the same conjugation site LC-K149C. However, significantly different activities were observed with B4/B2 (in bold) reaching 100% TGI (B1 < B3 < **B4/B2**), again in accordance with the intra-tumorally released MMAE concentration (B1 < B3 < **B4** < **B2**). Figure 2C depicts a series of aCD22-ss-maytansine (DMx) TDCs with different chemical modifications on disulfide linkers at sites LC-K149C (C1-C6) and S400C (C7-C8), respectively (Pillow et al., 2017). The efficacy ranking (C3 < C4/C5/C8 < **C1/C7/C2/C6**) was explained by the DMx concentration measured in the tumor (C3 < C4/C5/C8 < **C1** < **C7** < **C2** < **C6**) with C1/C7/C2/C6 (in bold) reaching 100% TGI. The ultimate payload release was therefore observed as the sequential/accumulative result of ADC stability in circulation and payload release kinetics as discussed previously.

Among the three sets of TDC analogs, complete TGI was observed with PBD-A3/A2, MMAE-B4/B2, DMx-C1/C7/C2/C6 TDCs despite the different intra-tumor payload concentration. In contrast to the previous aHER2-MMAE TDCs (Shen et al., 2012), the efficacy of TDCs A-C were not correlated with the stability/DAR profile or total antibody (Tab) exposure in plasma. However, the observed TDC efficacy results were consistently explained by the payload release and exposure in tumor above a certain threshold. In other words, the antitumor efficacy correlated with the intratumoral payload exposures with a “plateau” effect. For example, the estimated activity thresholds were 1 PBD/10^6^ bp (∼ 0.5 nM) for PBD TDCs, and 50 nM for MMAE TDCs and 13 nM for DMx TDCs, respectively (Figure 2). It is therefore important to deliver active payload to tumors in a manner that achieves a minimum or threshold level required for desired efficacy. For the three types of ADCs under investigation, the efficacy appears to be C_max_-driven given their highly bound microtubule binder and DNA alkylator payloads. Meanwhile TGI effects may plateau due to saturation of target (e.g., DNA) response to payloads over time. Linker design should be optimized to reach a balance between ADC stability and payload release kinetics and thus achieve the efficacious released payload in tumor cells above an apparent threshold. Payload concentration assessments in tumors can be utilized to predict the efficacy of ADCs in preclinical models.

## Coupling Integrated Linker Design With Dose Regimen Design for Optimal Efficacy and Toxicity Profiles

It is critical to deliver and release active payload components in the tumor cells above the threshold level required to drive efficacy. Importantly, a higher ADC dose is not always helpful to improve efficacy and, once the key threshold is reached, likely only increases the toxicity potential. Therefore, the integrated linker design should be coupled with dose regimen design to find the minimal efficacious dose. In mouse xenograft models, dose fraction studies were carried out to estimate the appropriate efficacious doses. TDC aHER2-HC-A118C-Me-SS-PBD achieved complete TGI with 1 × 1 mg/kg IV dose; while a 3 × 0.33 mg/kg (weekly) over 3 weeks regimen failed to reach the complete tumor inhibition endpoint in MMTV-HER2/Fo5 mice (Supplementary Figure S2A). TDC aCD22-LC-K149C-vc-PAB-MMAE exhibited better activity with 1 × 1.5 mg/kg compared to 3 × 0.5 mg/kg when tested in BJAB mice (Supplementary Figure S2B). Whereas the conjugate displayed similar efficacy with 1 × 3 mg/kg and 3 × 1 mg/kg doses sustained for over 7 weeks. TDC aCD22-LC-K149C-MCC-DM1 was tested in 3 dose-fraction groups with 3 dose regimens for reach group: 1.5 mg/kg total dose (3 × 0.5 mg/kg, 2 × 0.7 mg/kg, 1 × 1.5 mg/kg), 3 mg/kg total dose (3 × 1 mg/kg, 2 × 1.5 mg/kg, 1 × 3 mg/kg), 6 mg/kg total dose (2 × 3 mg/kg, 2 × 3 mg/kg, 1 × 6 mg/kg) (Supplementary Figure S2C). The larger total daily dose group resulted in better tumor inhibition although all tested dose regimens showed some activity. In addition, in each dose fraction group, the single dose displayed the best efficacy, indicating a payload C_max_-driven effect in tumors. In this situation, the single dose, when achieving complete TGI and better tumor inhibition than its other corresponding fraction dose regimens, can serve as the threshold dose for efficacy. In situations where the single-dose and multiple-dose fraction regimens result in similar efficacy, the minimal efficacious dose can be chosen based on the principal of delivering sufficient but not excessive payloads for optimal efficacy profiles and minimal toxicity potential. The MOAs of payload may play a role in determining which situations could be associated with investigated ADCs.

## Discussion

With the advent of diversified linker designs, ADC development has demonstrated rapid advancement in the last decade. Current site-specific technologies allow research endeavors to focus on exploration of various chemistries applied to linkage, linker cleavage and self-immolation. Herein, we provide our perspectives derived from our most recent studies in the past few years.

To achieve optimal PK profiles of active payloads and desired efficacy, modulation of both ADC stability and payload release are required. Good systemic ADC stability is desired so that linker-payloads are protected from hydrolysis in circulation. Meanwhile efficient linker cleavage and payload release (e.g., *via* self-immolation) are required to accumulate sufficient active payloads in tumors to reach the efficacy endpoints. The time to reach the threshold payload concentrations can depend on tumor types and ADC tissue penetration characteristics. Important modulating parameters to be incorporated into an integrated design approach are conjugation site, conjugation chemistry (e.g., maleimide vs disulfide linkers), linker length, cleavable/non-cleavable linkage and local linker steric hindrance. A more profound understanding is that biological activity correlated with the intratumoral payload concentration (Li et al., 2016; Zhang et al., 2018). Meanwhile a threshold payload concentration is required to support tumor stasis. In general, ADCs efficacy would depend on the combination effect of payload concentration and residence time in tumors. For the majority of ADCs in research, their payloads are highly bound with slow K_off_ values such as microtubule binder and DNA alkylators, and thus their efficacy appears to be payload C_max_-driven. Intra-tumor payload concentration assessments can be performed to establish a relationship with efficacy profiles and thus define the desired threshold drug concentration in tumors in preclinical models. Despite the uncertain translational outcomes from pre-clinical to clinical studies, the optimal efficacious dose choice would allow for a better estimation of therapeutic index. We therefore recommend integrated linker design approaches to target achieving the optimal intratumoral payload PK profiles by reaching above the threshold dose.

To achieve optimal efficacy and toxicity profiles, dose regimen hypotheses can be generated through dose-fractionation studies for different ADC designs. On one hand, there was an apparent range of increased payload concentrations resulting in improved tumor growth regression. Dose fractionation studies may be done to estimate dose regimens that offer maximal efficacy or tumor stasis. On the other hand, such linear relationship appeared to stop at some point followed by a “plateau” effect or E_max_ (Zhang et al., 2018). The observed “plateau” effect can be explained by payload C_max_, long payload retention, and target response saturation in tumors. Recognition of payload concentrations that result in efficacy “plateau” is important as the extra payload delivery to tumors does not improve efficacy and may cause toxicity due to the higher catabolite concentration in normal tissues. Optimal dose regimens can be selected beyond the current every-three-week cycle for antibody drugs by combining the dose fraction studies and prolonged payload PK in tumors as established in the relationship between intra-tumor payload concentration and efficacy. It is possible that for different types of ADCs different factors could play the more important roles. The antibody coadministration strategy could be applied to certain types of ADCs to improve the efficacy or lower the minimal efficacious dose by addressing poor ADC tissue penetration and distribution, e.g., payload being delivered to the same cells instead of others (Cilliers et al., 2018; Singh et al., 2020). In some cases, a different metric than TGI can be used to differentiate efficacy beyond complete response. Additional comprehensive investigations will help to better understand the ADC behaviors and their drivers and thus improve the therapeutic window.

In summary, an integrated linker design approach should be considered for optimal ADC design. Important parameters that can be employed include conjugation site, conjugation chemistry (e.g., maleimide vs. disulfide linkers), linker length, cleavable/non-cleavable linkage and local linker steric hindrance. The key point to be addressed is to achieve a balance between ADC stability and payload release for optimal intratumoral payload PK profiles and efficacy. In addition, coupling integrated linker design with dose fraction design is recommended to find the optimal dose regimens for desired efficacy and toxicity profiles.

## Data Availability

The original contributions presented in the study are included in the article/Supplementary Material, further inquiries can be directed to the corresponding authors.
