# Supplementary material for: Linker Design Impacts Antibody-Drug Conjugate Pharmacokinetics and Efficacy via Modulating the Stability and Payload Release Efficiency
Source: Front Pharmacol. 2021 Jun 23;12:687926. doi: 10.3389/fphar.2021.687926 (PMC8262647; doi:10.3389/fphar.2021.687926)
Supplement: Supplementary file 1 [file DataSheet1.docx]

Supplementary Material

# Supplementary Figures

**Supplementary Figure 1.**

Figure S1. Typical biotransformation next-gen TDCs via linker deconjugation, immolation and payload metabolism. Common catabolites and metabolites include unconjugated drugs or conjugated antibodies or drugs and naked antibodies.


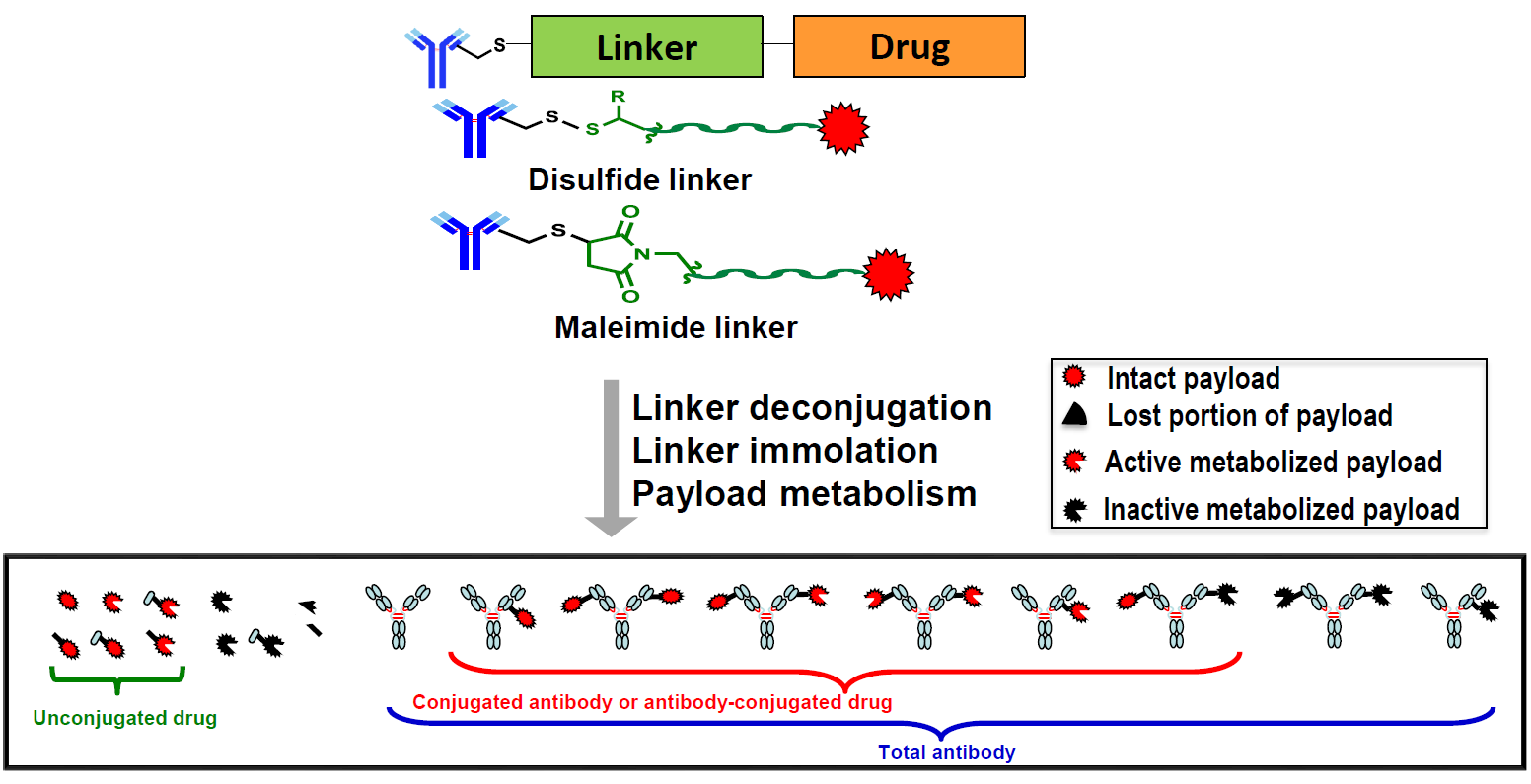


Figure S2. Dose-fractionation efficacy studies of PBD-, MMAE-, and DM1-containing ADCs D1, D2, and D3 in xenograft mice models. Xenograft mice (n=8 each treatment group) were dosed with a single intravenous dose or one-third or one-half of the dose every week three times or twice (A–C). Tumors in mice were measured one to two times a week throughout the studies. The tumor volumes were plotted as a mean tumor volume S.E.M. of each group over time. Reproduced from (Zhang et al., 2019). Copyright 2019 American Society for Pharmacology and Experimental Therapeutics.
